# SNHG16 promotes tumorigenesis and cisplatin resistance by regulating miR-338-3p/PLK4 pathway in neuroblastoma cells

**DOI:** 10.1186/s12935-020-01291-y

**Published:** 2020-06-12

**Authors:** Zhaoying Xu, Yongfa Sun, Danfeng Wang, Huifang Sun, Xiaojun Liu

**Affiliations:** Department of Pediatrics, Luoyang Central Hospital Affiliated To Zhengzhou University, No. 288 Zhongzhou Middle Road, Luoyang, 471000 Henan China

**Keywords:** SNHG16, miR-338-3p, PLK4, Cisplatin resistance, Neuroblastoma

## Abstract

**Background:**

Long noncoding RNA small nucleolar RNA host gene 16 (lncRNA SNHG16) has been revealed to be involved in the tumorigenesis of neuroblastoma. However, the role of SNHG16 in regulating cisplatin sensitivity in neuroblastoma remains largely unknown.

**Methods:**

The expression of SNHG16, microRNA (miR)-338-3p and polo-like kinase 4 (PLK4) mRNA was measured using quantitative real-time polymerase chain reaction. The protein levels of PLK4, multidrug resistance protein 1 (MRP1), multidrug-resistance gene 1-type p-glycoprotein (P-gp) and phosphoinositide 3-kinase (PI3K)/protein kinase B (AKT) pathway-related proteins were detected by Western blot. The half maximal inhibitory concentration (IC50) value, cell proliferation, migration and invasion were analyzed using Cell Counting Kit-8 assays or Transwell assay. Apoptotic cells were measured by Flow cytometry. The interaction between miR-338-3p and SNHG16 or PLK4 was confirmed by dual-luciferase reporter and RNA immunoprecipitation assay. In vivo experiments were conducted through the murine xenograft model.

**Results:**

SNHG16 was up-regulated, while miR-338-3p was down-regulated in cisplatin-resistant neuroblastoma tissues and cells. SNHG16 silencing weakened cisplatin resistance, reflected by the reduction of IC50 value, down-regulation of MRP-1 and P-gp protein expression, suppression of proliferation, migration and invasion, as well as enhancement of apoptosis in SNHG16 deletion cisplatin-resistant neuroblastoma cells. Besides that, SNHG16 could regulate PLK4 expression by sponging miR-338-3p and SNHG16/miR-338-3p/PLK4 axis could affect the activation of PI3K/AKT pathway in cisplatin-resistant neuroblastoma cells. MiR-338-3p inhibition attenuated SNHG16 deletion-mediated impairment on cisplatin resistance and PLK4 overexpression reversed the decrease of cisplatin-resistance induced by miR-338-3p re-expression. Furthermore, SNHG16 knockdown contributed to the anti-tumor effect of cisplatin in neuroblastoma in vivo.

**Conclusion:**

SNHG16 contributed to the tumorigenesis and cisplatin resistance in neuroblastoma possibly through miR-338-3p/PLK4 pathway, indicating a novel insight for overcoming chemoresistance in neuroblastoma patients.

## Background

Neuroblastoma is the most common extracranial tumor in children and infants, accounting for about 6–10% of pediatric malignancies, and is responsible for 15% cancer-associated mortality in children [1, 2]. With the progression of multimodal therapy, such as high-dose myeloablative chemotherapy with autologous hematopoietic stem cell transplantation and immunotherapy, many neuroblastoma patients are cured. However, there are still survivors of neuroblastoma suffering from severe side effects and drug resistance [3, 4]. Thus, further investigations to identify detective and prognostic biomarkers, as well as develop low-toxic treatment strategies are required for managing the survival of neuroblastoma.

Long noncoding RNAs (lncRNAs) are commonly type transcripts with over 200 nucleotides in size and are not translated into protein. It has been documented that lncRNAs can control gene expression at the epigenetic, transcription and post-transcription levels and also participate in regulating multiple biological processes, including cellar proliferation, apoptosis, metastasis, and epithelial-to-mesenchymal transition (EMT) [5–8]. Besides, it was also revealed that several abnormally expressed lncRNAs can mediate chemoresistance in cancers [9]. All these evidence suggests lncRNAs are potential diagnostic and prognostic targets for malignancies. LncRNA small nucleolar RNA host gene 16 (SNHG16) is a cancer-related lncRNA, which was found to be elevated in diverse cancers, such as breast cancer [10], gastric cancer [11], osteosarcoma [12], and hepatocellular carcinoma [13], and acted as an oncogene to contribute to the drug resistance and development of cancers. In neuroblastoma, only Yu et al. indicated SNHG16 was up-regulated and highly expressed SNHG16 predicted poor prognosis; besides that, SNHG16 deletion suppressed cell tumorigenesis, thus regulating neuroblastoma development [14]. However, the exact mechanisms underlying the chemoresistant role of SNHG16 in neuroblastoma remain largely vague.

MicroRNAs (miRNAs) are another type of non-coding RNAs which affect gene expressions in many processes, thereby regulating mRNA degradation or translational suppression [15]. So far, there are a growing amount of studies uncover the aberrant expression of some miRNAs in neuroblastoma and dysregulated miRNAs mediate the modulation of diverse biological processes in neuroblastoma cells to regulate the initiation and progression of neuroblastoma [16–18]. MiR-338-3p is a well-documented miRNA, which has been revealed to perform anti-tumor effects in a variety of cancers, including hepatocellular carcinoma, non-small cell lung cancer, renal cell carcinoma, and neuroblastoma [19–22]. Polo like kinase 4 (PLK4) is a kinase, whose activity is important for centriole duplication, and plays a well-characterized effect on diverse cell cycle processes, thus controlling proper growth and division of cells [23]. Previous study indicated PLK4 was up-regulated in neuroblastoma and deregulated PLK4 was associated with the tumorigenesis in neuroblastoma cells [24]. Thus, deeper investigations on PLK4 and miR-338-3p in neuroblastoma drug resistance are required.

Here, this work aimed to explore the function of SNHG16 and miR-338-3p in neuroblastoma cell drug resistance, investigated the relationship between SNHG16 and miR-338-3p, as well as identify the means by which it mediating PLK4 to affect neuroblastoma drug resistance.

## Materials and methods

### Patients and specimens

A total of 76 neuroblastoma tissues were obtained from patients who underwent surgical resection at Luoyang Central Hospital Affiliated To Zhengzhou University. All patients were diagnosed by two independent pathologists and only received cisplatin-based neo-adjuvant chemotherapy prior to surgery. In addition, 76 neuroblastoma patients were classified into cisplatin-resistant and -sensitive depending on the sensitivity to cisplatin: cisplatin-sensitive group (Sensitivity, tumor remission after 6 cycles of chemotherapy, N = 36) and cisplatin-resistant group (Resistance, tumor stabilization or progression after 6 cycles of chemotherapy, N = 40). Besides, the clinicopathological parameters of all subjects, including age, gender, INSS staging, Tumor size, and metastasis, were collected. Follow-up was performed regularly every 3 months in the first 2 years after surgery, and it was reduced to once every 6 months from the third year onward. The last follow-up was carried out in January 2019. Samples were immediately stored at − 80 °C until used.

### Cell culture and transfection

Neuroblastoma SK-N-AS (doubling time: 50 h) and SK-N-SH cell lines (doubling time: 40 h) were purchased from American Type Culture Collection (ATCC, Manassas, VA, USA) and cultured in Dulbecco’s modifed Eagle’s medium (DMEM; Gibco, Carlsbad, CA, USA) harboring with 10% fetal bovine serum, 100 U/mL penicillin and 100 µg/mL streptomycin at 37 °C with 5% CO_2_. SK-N-AS and SK-N-SH cells were exposed stepwise increasing concentrations of cisplatin over 6 months to generate cisplatin-resistant cells, named SK-N-AS-R and SK-N-SH-R (bulk populations). The initial IC50 of SK-N-AS was 1.2 μM and final concentration was 24 μM cisplatin concentration. The initial cisplatin concentration of SK-N-SH was 33 μM and the final IC50 was 180 μM cisplatin concentration.

The miR-338-3p mimic (miR-338-3p), miR-338-3p inhibitor (in-miR-338-3p), and their corresponding negative control (miR-NC and in-miR-NC) were purchased from RIBOBIO (Guangzhou, China). The short hairpin RNA (shRNA) targeting SNHG16 (sh-SNHG16, 5′-GGAATGAAGCAACTGAGATTT-3′), shRNA scramble control (sh-NC, 5′-TTCTCCGAACGTGTCACGTTT-3′), a pooled sequence of small interfering RNA (siRNA) targeting SNHG16 (si-SNHG16, 5′-GGAAUGAAGCAACUGAGAUUU-3′; 5′-ACUUUAGAGGAACAAUUAGCA-3′), siRNA negative control (si-NC, 5′-UUCUCCGAACGUGUCACGUTT-3′), empty vector (pcDNA), pcDNA-SNHG16 overexpression vector (SNHG16), pcDNA- PLK4 overexpression vector (PLK4) were synthesized by Genepharma (Shanghai, China). All miRNAs or vectors were transfected into SK-N-AS-R and SK-N-SH-R cells using Lipofectamine™ 2000 transfection reagent (Invitrogen, Carlsbad, CA, USA) for 48 h.

### Quantitative real-time polymerase chain reaction (qRT-PCR)

TRIzol reagent (Invitrogen) was used to exact total RNA according to the standard procedure. Complementary DNA (cDNA) was synthesized using a High Capacity cDNA Reverse Transcription Kit (Qiagen, Valencia, CA, USA), and then quantitative PCR was carried out with SYBR Premix Ex Taq (Qiagen). Fold changes were analyzed by 2^−∆∆Ct^ method and normalized by glyceraldehyde 3-phosphate dehydrogenase (GADPH) or U6 small nuclear B noncoding RNA (U6). The specific primer sequences were listed as follows: SNHG16: F 5′-GCAGAATGCCATGGTTTCCC-3′, R 5′-GGACAGCTGGCAAGAGACTT-3′; PLK4: F 5′-GACACCTCAGACTGAAACCGTAC-3′, R 5′-GTCCTTCTGCAAATCTGGATGGC-3′; miR-338-3p: F 5′-TGCGGTCCAGCATCAGTGAT-3′, R 5′-CCAGTGCAGGGTCCGAGGT-3′. GADPH: F 5′-GATATTGTTGCCATCAATGAC-3′, R 5′-TTGATTTTGGAGGGATCTCG-3′; U6: F 5′-CTCGCTTCGGCAGCACA-3′, R 5′-ACGCTTCACGAATTTGCGT-3′.

### Western blot

Proteins were extracted using RIPA lysis buffer (Beyotime, Beijing, China) and quantified by bicinchoninic acid method following the standard protocol. After blocked with 1% non-fat milk, immunoblot assays were performed using specific primary antibodies PLK4 (1:500, ab2642, Abcam, Cambridge, MA, USA), MRP1 (1:1000, ab233383, Abcam), P-gp (1:400, ab103477, Abcam,), phosphorylated (p)-phosphoinositide 3-kinase (p-PI3K) (1:1000, ab182651, Abcam), PI3K (1:1000, ab40776, Abcam), p-protein kinase B (p-AKT) (1:1000, 9271, Cell Signaling Technology (CST), Boston, MA, USA), AKT (1:1000, 9272, CST), N-cadherin (1:1000, ab18203, Abcam), E-cadherin (1:1000, ab15148, Abcam), (PCNA) (1:5000, ab29, Abcam) as well as β-actin (1:1000, 4967, CST), and followed by interaction with secondary HRP-conjugated antibody (1:1000, ab9482, Abcam). Finally, protein signals were examined using a chemiluminescence chromogenic substrate (Beyotime) method.

### Cells viability and proliferation assay

Cell counting kit-8 (CCK-8) assay was used to analyze cell viability and proliferation. Cells (5000 per well) were seeded into 96-well plates overnight and then incubated with 10 μL CCK-8 solution for 2 h. Subsequently, the absorbance at a wavelength of 450 nm was determined by a microplate reader (Bio-Rad, Hercules, CA, USA) in the indicated time. Besides that, the IC50 value of drugs was assessed according to the relative survival curve.

### Cells migration and invasion assays

For migration assay, cells in serum-free DMEM were planted in the top chambers. 500 μL DMEM mixed with 10% FBS was added into the lower chambers. After incubation for 24 h at 37 °C, cells on the lower face of the membrane were fixed and stained. Finally, migrated cells in five randomly selected fields were counted with a microscope (Olympus, Tokyo, Japan). For invasion assay, the matrigel (BD Biosciences, San Jose, CA, USA) was pre-coated in the chamber membranes and other philosophy of measurement was similar to the steps of cell migration.

### Cells apoptosis assay

In brief, cells were resuspended with binding buffer, followed by staining with 10 μL fluorescein isothiocyanate (FITC) annexin V and propidium iodide (PI) (BD Biosciences). Finally, the apoptotic cells were analyzed using a flow cytometer (FACScan; BD Biosciences, Shanghai, China).

### Dual-luciferase reporter assay

The SNHG16 mRNA and PLK4 3′-UTR containing wild-type (WT) or mutant (MUT) binding sequences of miR-338-3p were cloned into the pmiR-RB-Report (Promega, Shanghai, China), respectively. After that, SK-N-AS-R and SK-N-SH-R cells were co-transfected with these construct luciferase reporter vectors and miR-338-3p mimics or miR-NC using Lipofectamine™ 2000 (Invitrogen). After 48 h of transfection, a dual luciferase assay kit (Promega) was used to detect the luciferase activity.

### RNA immunoprecipitation (RIP) assay

RIP assay was performed using Magna RNA immunoprecipitation kit (Millipore, Billerica, MA, USA). Neuroblastoma cells were lysed, and then 100 μL lysate was incubated with magnetic beads coating with anti-Ago2 (Millipore) or IgG antibody (Abcam). Finally, immunoprecipitated RNA was extracted and purified RNA was examined by qRT-PCR using the 7500 Real‐Time PCR system (Thermo Fisher Scientific, Waltham, MA, USA).

### Xenograft mice assay in vivo

Five-week-old athymic BALB/c mice (N = 12) were used to establish xenograft models according to the guidelines permitted by the Animal Research Committee of Luoyang Central Hospital Affiliated to Zhengzhou University. SK-N-AS-R cells stably infected with lentivirus-(lenti)-sh-SNHG16 or lenti-sh-NC were subcutaneously inoculated into the flanks of the nude mice, followed by intravenously injection with cisplatin (3 mg/kg) every 7 days after inoculation for 7 days. Subsequently, tumor volume was calculated every 7 days. After 28 days, all mice were killed and tumor masses were weighed and used for further molecular analysis.

### Statistical analysis

Data from three independent experiments was expressed as the mean ± standard deviation (SD) and analyzed with GraphPad Prism 7 (GraphPad Inc., La Jolla, CA, USA). Statistical differences were analyzed by double-sided Student’s *t* test (two groups) or one-way analysis of variance (ANOVA) followed by Tukey post hoc test (more than two groups) as appropriate. The correlation analysis was analyzed using Spearman’s correlation test. *P *< 0.05 indicated statistically significant.

## Results

### SNHG16 is up-regulated and miR-338-3p is down-regulated in cisplatin-resistant neuroblastoma tissues and cells

**Fig. 1:**
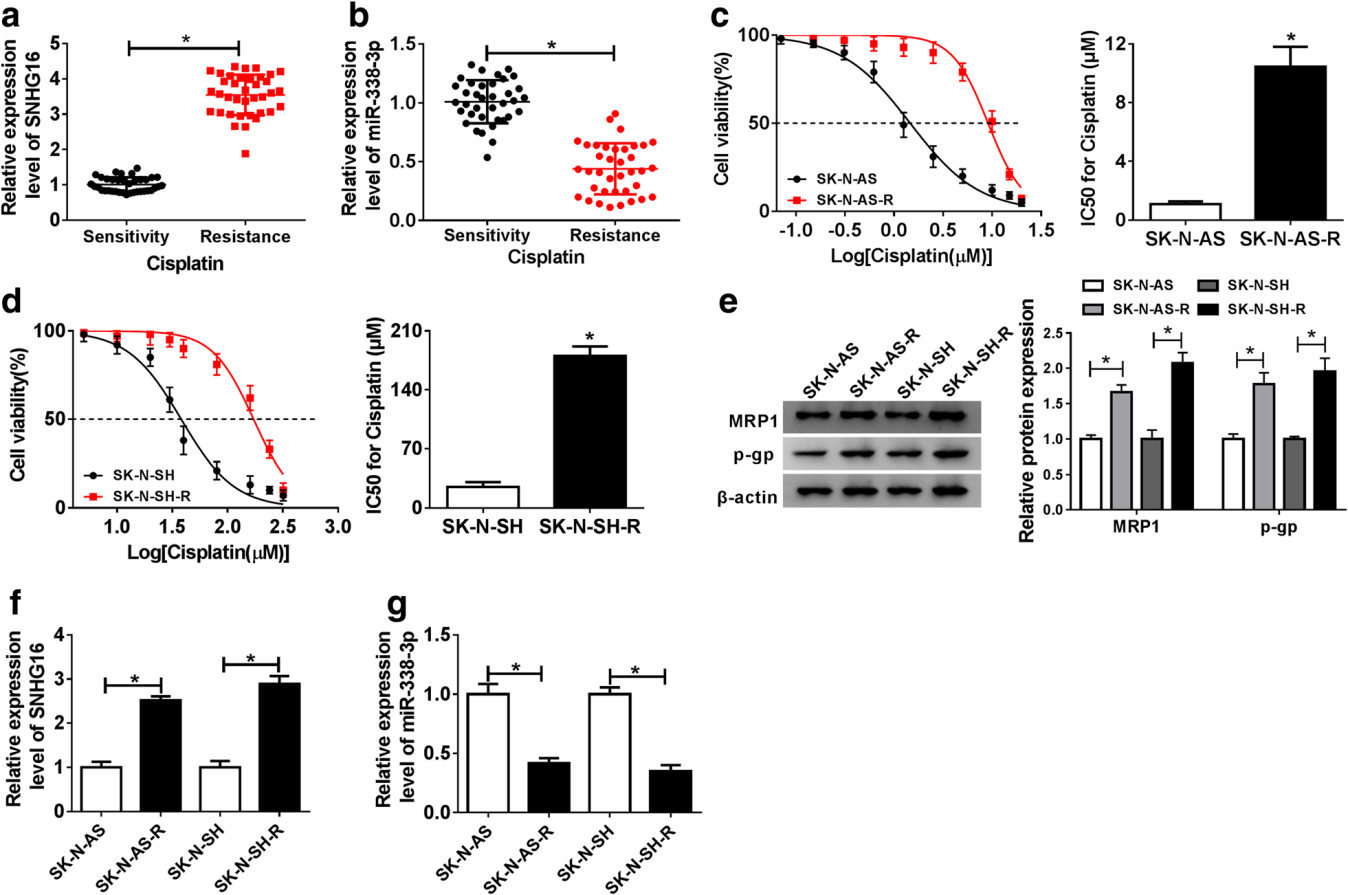
SNHG16 is up-regulated and miR-338-3p is down-regulated in cisplatin resistant neuroblastoma tissues and cells. **a**, **b** The expression of SNHG16 and miR-338-3p was detected using qRT-PCR in cisplatin resistant and sensitive neuroblastoma tumor tissue. **c**, **d** The IC50 value for cisplatin was calculated by CCK-8 assay in SK-N-AS-R and SK-N-SH-R cells. **e** Western blot analysis of the levels of MRP1 and p-gp protein in cisplatin-resistant neuroblastoma cell lines SK-N-AS-R and SK-N-SH-R and parental SK-N-AS and SK-N-SH was performed. **f**, **g** The expression of SNHG16 and miR-338-3p was detected using qRT-PCR in cisplatin-resistant neuroblastoma cell lines (SK-N-AS-R and SK-N-SH-R) and corresponding parental neuroblastoma cell lines (SK-N-AS and SK-N-SH). The same experiment was repeated three times, and the average was taken. *P < 0.05

Previous study has showed that SNHG16 was up-regulated in neuroblastoma, thus, we detected the expression of SNHG16 in neuroblastoma tissues which were divided into cisplatin-sensitive group (Sensitivity, N = 36) and cisplatin-resistant group (Resistance, N = 40). qRT-PCR analysis indicated SNHG16 was significantly up-regulated in the Resistance group compared to the Sensitivity group (Fig. 1a). Then patients were divided into two groups based on the median level of SNHG16, the median survival time of patients with high SNHG16 was remarkably shorter than that with low SNHG16 expression (*P* = 0.003, Table 1); moreover, higher SNHG16 expression was correlated with INSS staging (*P* = 0.011) and metastasis (*P* = 0.028) (Table 1). These results suggested SNHG16 expression was is dysregulated in neuroblastoma and might be associated with cisplatin resistance.

**Table 1:** Correlation between SNHG16 expression and neuroblastoma clinicopathological parameters

Parameters	n	SNHG16		*P*
		High(n = 38)	Low(n = 38)	
Age(years)				
< 5	52	28	24	0.324
≥ 5	24	10	14	
Gender				
Female	40	19	21	0.646
Male	36	19	17	
INSS staging				
1–2	41	13	28	0.011*
3–4	29	20	9	
4 s	6	5	1	
Tumor size (cm)				
≥ 7	28	16	22	0.054
< 7	48	22	26	
Metastasis				
Yes	25	17	8	0.028^*^
No	51	21	30	
Median survival time (months)		32.05 ± 8.54	38.6 ± 9.86	0.003*

Note: *P < 0.05

In addition, we also observed that miR-338-3p expression was down-regulated in the Resistance group compared to the Sensitivity group (Fig. 1b). Subsequently, cisplatin resistant cell models in vitro were established by exposing SK-N-AS and SK-N-SH cells to stepwise increasing concentrations of cisplatin over 6 months. The value of IC50 demonstrated that SK-N-AS-R and SK-N-SH-R cells were remarkable more resistant to cisplatin than these cisplatin-sensitive cells (SK-N-AS and SK-N-SH) (Fig. 1c, d), meanwhile, western blot analysis showed the levels of resistant protein MRP1and p-gp were elevated in SK-N-AS-R and SK-N-SH-R cells compared with parental SK-N-AS and SK-N-SH cells (Fig. 1e). All the data confirmed the successful establishment of cisplatin-resistant cells. Afterwards, the elevation of SNHG16 and decrease of miR-338-3p in cisplatin-resistant neuroblastoma cell lines (SK-N-AS-R and SK-N-SH-R) were also observed (Fig. 1f, g). These data indicated that abnormal expression of SNHG16 or miR-338-3p might related to cisplatin resistance in neuroblastoma.

### SNHG16 deletion inhibits cell cisplatin resistance and malignant phenotypes in neuroblastoma

**Fig. 2:**
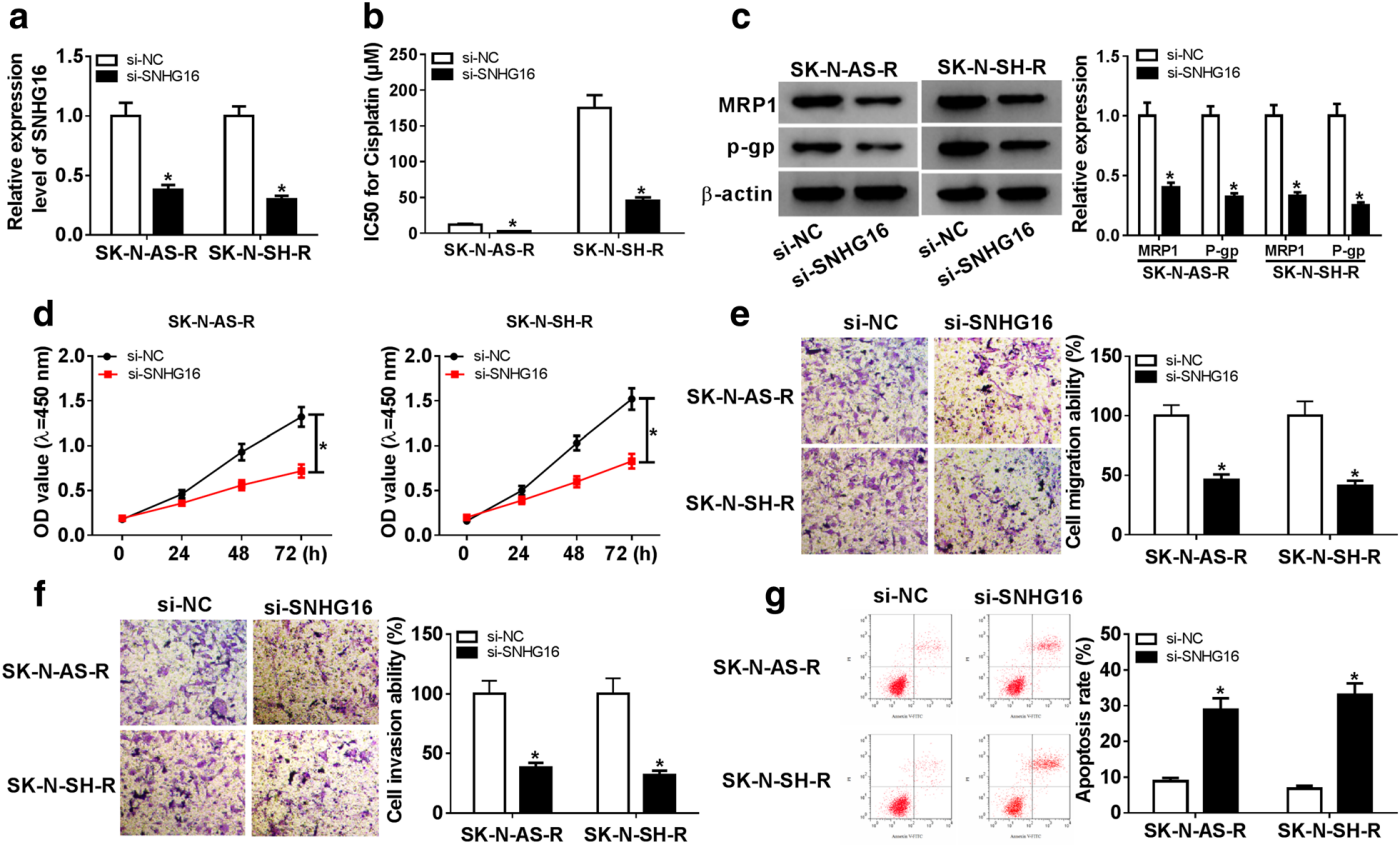
SNHG16 deletion inhibits cell cisplatin resistance and malignant phenotypes in neuroblastoma. SNHG16 was silenced in SK-N-AS-R and SK-N-SH-R cells using siRNA sequences targeting SNHG16. **a** The relative expression of SNHG16 was measured using qRT-PCR. **b** The IC50 value for cisplatin was assessed by CCK-8 assay. **c** The expressions of drug-resistance associated proteins MRP1 and P-gp were examined by western blot assay. **d** The CCK-8 assay was performed to detect cell proliferation. **e**, **f** Transwell assay was used to determine cell migration and invasion ability. **g** Cell apoptosis was analyzed using Flow cytometric analysis. The same experiment was repeated three times, and data represented as the average of three independent replicates. *P < 0.05

To investigate the biological functions of SNHG16 in cisplatin resistance of neuroblastoma, SNHG16 was silenced in SK-N-AS-R and SK-N-SH-R cells using siRNA sequences targeting SNHG16. As expected, the level of SNHG16 was greatly down-regulated in SK-N-AS-R and SK-N-SH-R cells (Fig. 2a). Subsequently, CCK-8 assay indicated that SNHG16 deletion led SK-N-AS-R and SK-N-SH-R cells sensitive to cisplatin, reflected by the decrease of IC50 value and expression of drug-resistance associated protein expression MRP-1 and P-gp in SK-N-AS-R and SK-N-SH-R cells (Fig. 2b, c). Furthermore, SNHG16 silence also inhibited the proliferation of SK-N-AS-R and SK-N-SH-R cells (Fig. 2d). After that, we also found knockdown of SNHG16 suppressed migration and invasion but induced apoptosis in SK-N-AS-R and SK-N-SH-R cells (Fig. 2e–g). Additionally, it was proved that knockdown of SNHG16 decreased the levels of PCNA and N-cadherin, while increased the level of E-cadherin in SK-N-AS-R and SK-N-SH-R cells (Additional file 1: Fig. S1A). Taken together, knockdown of SNHG16 inhibited tumorigenesis and cisplatin resistance in cisplatin-resistant neuroblastoma cells.

### SNHG16 is a sponge of miR-338-3p

**Fig. 3:**
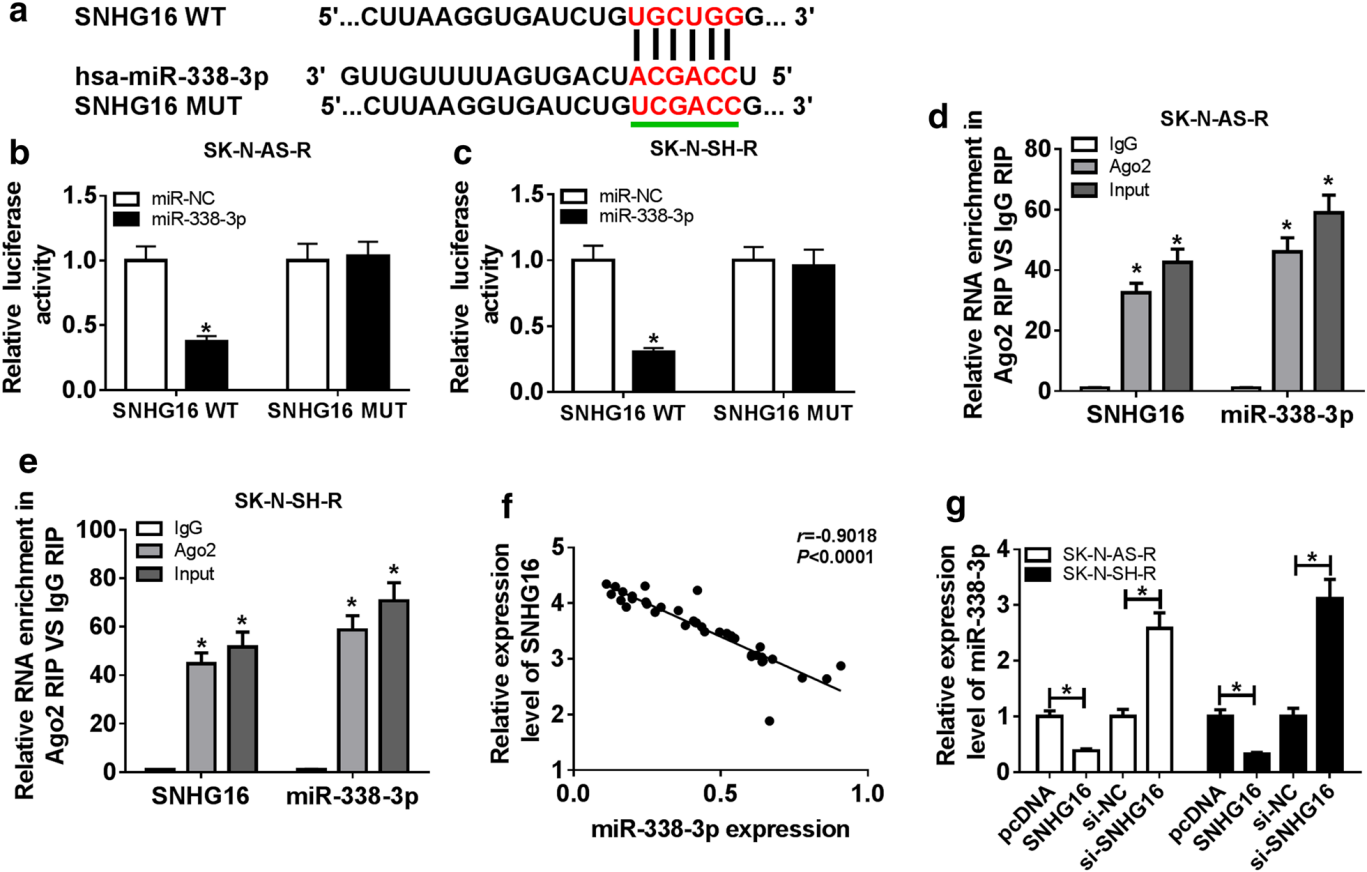
SNHG16 is a sponge of miR-338-3p. **a** The putative binding site between SNHG16 and miR-338-3p was predicted. **b**–**e** The interaction between SNHG16 and miR-338-3p was confirmed by the dual-luciferase reporter assay and RIP assay in SK-N-AS-R and SK-N-SH-R cells, respectively. **f** The correlation between SNHG16 and miR-338-3p was analyzed using Spearman’s correlation test. **g** The relative expression of miR-338-3p in SK-N-AS-R and SK-N-SH-R cells transfected with pcDNA, SNHG16, si-NC, or si-SNHG16 was detected by qRT-PCR (N = 3). N means that triplicate individual experiments were performed, and the average was taken. *P < 0.05

To elucidate the biological pathway underlying SNHG16 in carcinogenesis and cisplatin resistance in neuroblastoma, the potential miRNA targets of SNHG16 were predicted using LncBase Predicted v.2 program, and many of miRNAs were identified to have binding sequences in SNHG16, among these miRNAs, miR-338-3p was selected for further exploration owing to its anticancer effects on neuroblastoma cells [22] (Fig. 3a). To confirm this prediction, the dual-luciferase reporter assay was performed and results showed overexpressed miR-338-3p reduced the luciferase activity of the SNHG16 WT reporter vector but not SNHG16 MUT reporter vector in SK-N-AS-R and SK-N-SH-R cells (Fig. 3b, c). Meanwhile, the RIP assay showed the significant enrichment of SNHG16 and miR-338-3p in SK-N-AS-R and SK-N-SH-R cells after Ago2 RIP (Fig. 3d, e). Moreover, a negative correlation between miR-338-3p and SNHG16 was observed (Fig. 3f) and co-expression analysis showed the expression of miR-338-3p was inhibited by SNHG16 overexpression, but was promoted by SNHG16 knockdown in SK-N-AS-R and SK-N-SH-R cells (Fig. 3g). Collectively, these results indicated SNHG16 was a sponge of miR-338-3p and negatively regulated miR-338-3p expression.

### SNHG16 deletion impairs cell cisplatin resistance and malignant phenotypes by sponging miR-338-3p in neuroblastoma

**Fig. 4:**
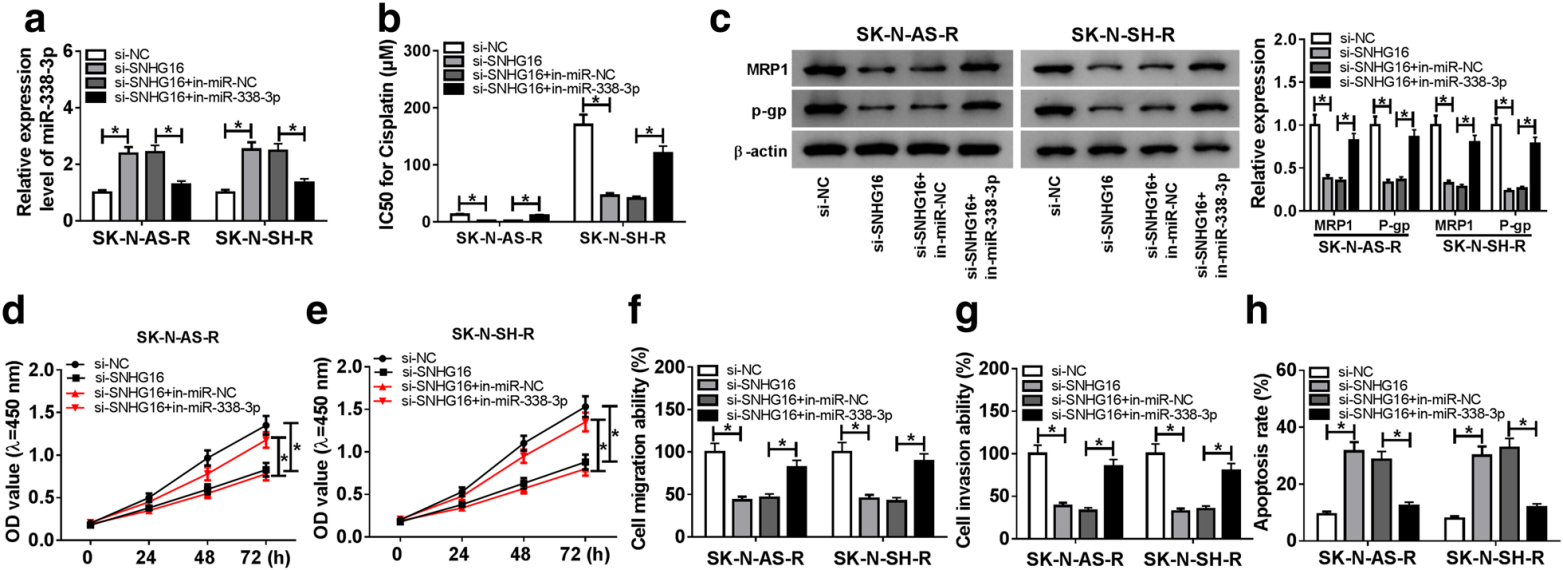
SNHG16 deletion impairs cell cisplatin resistance and malignant phenotypes by sponging miR-338-3p in neuroblastoma. SK-N-AS-R and SK-N-SH-R cells were transfected with si-NC, si-SNHG16, si-SNHG16 + in-miR-NC, si-SNHG16 + in-miR-338-3p. **a** The relative expression of miR-338-3p was measured using qRT-PCR. **b** The IC50 value for cisplatin was assessed by CCK-8 assay. **c** Western blot assay was used to detect the expression of drug-resistance associated proteins MRP1 and P-gp. **d**, **e** Cell proliferation was examined using CCK-8 assay. **f**, **g** Transwell assay was used for the detection of cell migration and invasion ability. **h** Cell apoptosis was analyzed using Flow cytometric analysis. Each experiments were repeated three times independently, and the average was taken. *P < 0.05

Based on the relationship between SNHG16 and miR-338-3p, we further gained insight into whether miR-338-3p was involved in the action of si-SNHG16 in cisplatin resistance and carcinogenesis in neuroblastoma cells. SK-N-AS-R and SK-N-SH-R cells were transfected with si-NC, si-SNHG16, si-SNHG16 + in-miR-NC, si-SNHG16 + in-miR-338-3p, and efficiencies of transfection were confirmed by qRT-PCR (Fig. 4a). Immediately, data of rescue assay showed the introduction of miR-338-3p inhibitor greatly attenuated si-SNHG16-induced drug sensibility to cisplatin (Fig. 4b), reduction of MRP-1 and P-gp protein expression (Fig. 4c), the suppression of proliferation (Fig. 4d, e), migration and invasion (Fig. 4f, g), as well as the promotion of apoptosis (Fig. 4h) in SK-N-AS-R and SK-N-SH-R cells. In addition, western blot analysis showed silencing miR-338-3p reversed si-SNHG16-induced elevation of E-cadherin, and down-regulation of PCNA and N-cadherin in SK-N-AS-R and SK-N-SH-R cells (Additional file 1: Fig. S1 A). Thus, we demonstrated that SNHG16 deletion weakened cisplatin resistance and suppressed cell progression by sponging miR-338-3p in neuroblastoma.

### SNHG16 indirectly regulates PLK4 expression by interacting with miR-338-3p

**Fig. 5:**
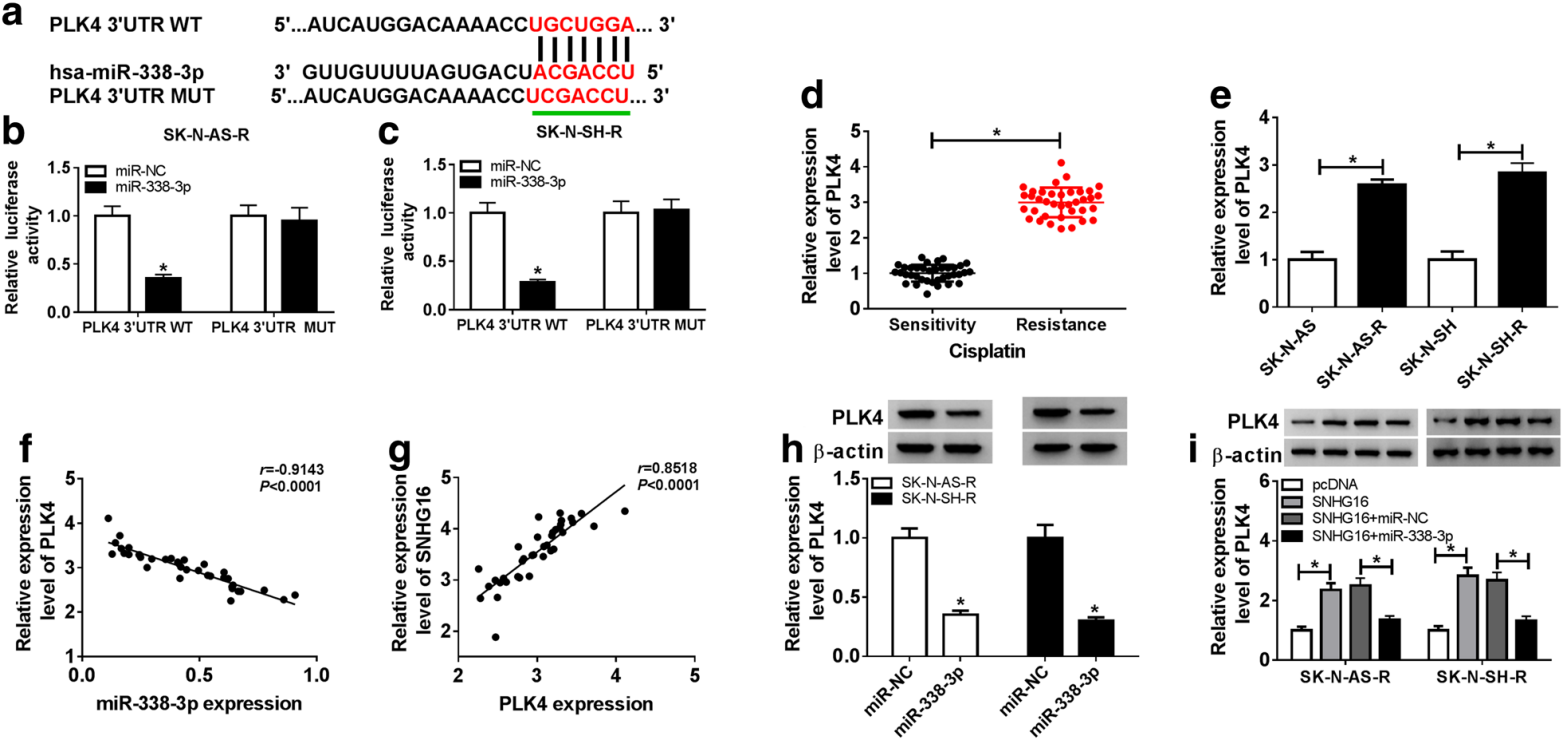
SNHG16 indirectly regulates PLK4 expression by interacting with miR-338-3p. **a** The putative binding site between PLK4 and miR-338-3p was listed. **b**–**c** The interaction between PLK4 and miR-338-3p was confirmed by dual-luciferase reporter assay in SK-N-AS-R and SK-N-SH-R cells. **d**, **e** The expression of PLK4 was detected using qRT-PCR in cisplatin-resistant neuroblastoma tissues and cell lines (N = 3). **f**, **g** The correlation between PLK4 and miR-338-3p or SNHG16 was analyzed using Spearman’s correlation test. **h** The protein expression of PLK4 in SK-N-AS-R and SK-N-SH-R cells transfected with miR-NC, miR-338-3p was measured using western blot (N = 3). **i** The level of PLK4 protein was examined by western blot in SK-N-AS-R and SK-N-SH-R cells transfected with pcDNA, SNHG16, SNHG16 + miR-338-3p, or SNHG16 + miR-NC (N = 3). N means that triplicate individual experiments were performed, and the average was taken. *P < 0.05

We further explored the potential molecular mechanisms by which SNHG16/miR-338-3p axis affecting cisplatin resistance and tumorigenesis in neuroblastoma cells. MicroT-CDS predictive tool was performed to search for the potential targets of miR-338-3p. As shown in Fig. 5a, PLK4 harbored putative complementary sequences for miR-338-3p, and was selected for further exploration due to its association with the tumorigenesis of neuroblastoma cells [24]. Subsequent dual luciferase reporter assay demonstrated that miR-338-3p overexpression effectively reduced the luciferase activity of the PLK4 WT reporter vector but not PLK4 MUT reporter vector in SK-N-AS-R (Fig. 5b) and SK-N-SH-R (Fig. 5c) cells. Subsequently, the level of PLK4 was detected and PLK4 was significantly increased in cisplatin-resistant neuroblastoma tissues and cells (Fig. 5d, e). Moreover, a negative correlation between miR-338-3p and PLK4, and a positive correlation between SNHG16 and PLK4 were determined (Fig. 5f, g). Besides that, western blot analysis indicated that miR-338-3p negatively regulated PLK4 expression (Fig. 5h), and miR-338-3p re-expression could reverse SNHG16 overexpression-triggered a substantial increase of PLK4 protein expression in SK-N-AS-R and SK-N-SH-R cells (Fig. 5i). Therefore, these data suggested SNHG16 indirectly regulated PLK4 expression by sponging miR-338-3p in cisplatin-resistant neuroblastoma cells.

### PLK4 overexpression reverses miR-338-3p re-expression-mediated inhibition on cisplatin resistance and tumorigenesis in neuroblastoma cells

**Fig. 6:**
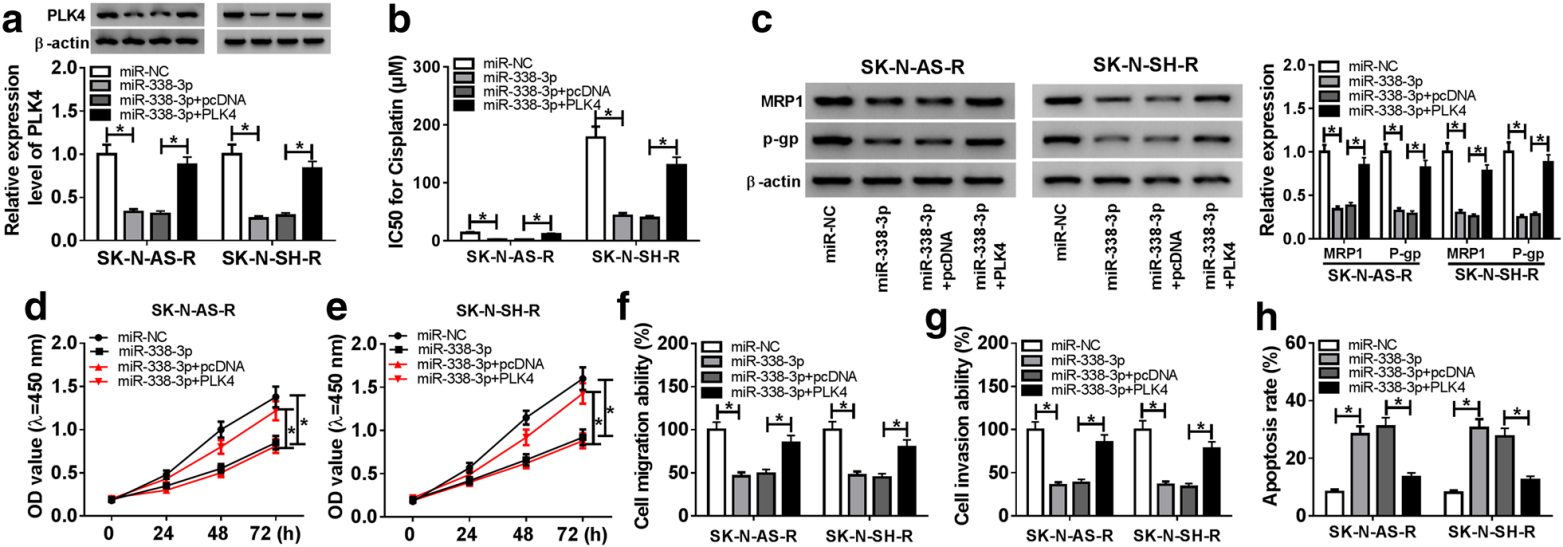
PLK4 overexpression reverses miR-338-3p re-expression-mediated inhibition on cisplatin resistance and tumorigenesis in neuroblastoma cells. SK-N-AS-R and SK-N-SH-R cells were transfected with miR-NC, miR-338-3p, miR-338-3p + pcDNA, or miR-338-3p + PLK4. **a** Western blot was used to detect the expression of PLK4. **b** The IC50 value for cisplatin was calculated by CCK-8 assay. **c** The expressions of drug-resistance associated proteins MRP1 and P-gp were examined by western blot assay. **d**, **e** The CCK-8 assay was performed to detect cell proliferation. **f**, **g** Transwell assay was used to determine cell migration and invasion ability. **h** The apoptosis rate was analyzed using Flow Cytometry. Each experiment was performed for three times, and results represented as the average of three independent replicates. *P < 0.05

According to the regulatory network of miR-338-3p/PLK4, we wanted to investigate whether miR-338-3p/PLK4 axis was responsible for cell cisplatin resistance and tumorigenesis in neuroblastoma. SK-N-AS-R and SK-N-SH-R cells were transfected with miR-NC, miR-338-3p, miR-338-3p + pcDNA, miR-338-3p + PLK4 and efficiencies of transfection were confirmed by western bolt (Fig. 6a). Immediately, we found the value of IC50 was decreased by miR-338-3p restoration, but was rescued by following PLK4 up-regulation (Fig. 6b). Western blot indicated overexpressed PLK4 could reverse miR-338-3p re-expression-mediated reduction of MRP-1 and P-gp protein expression (Fig. 6c). Besides that, we also demonstrated that highly expressed PLK4 could partially overturn miR-338-3p restoration-induced inhibition on SK-N-AS-R and SK-N-SH-R cell proliferation (Fig. 6d, e), migration and invasion (Fig. 6f, g), as well as promotion on cell apoptosis (Fig. 6h). Furthermore, miR-338-3p overexpression decreased the levels of PCNA and N-cadherin, while increased the level of E-cadherin in SK-N-AS-R and SK-N-SH-R cells, while this condition was abolished by PLK4 up-regulation (Additional file 1: Fig. S1B). Altogether, we confirmed that miR-338-3p could enhance the sensitivity of cisplatin-resistant neuroblastoma cells to cisplatin and suppressed cell tumorigenesis by regulating PLK4.

### SNHG16/miR-338-3p/PLK4 axis can regulate the activation of PI3K/AKT pathway in neuroblastoma cells

**Fig. 7:**
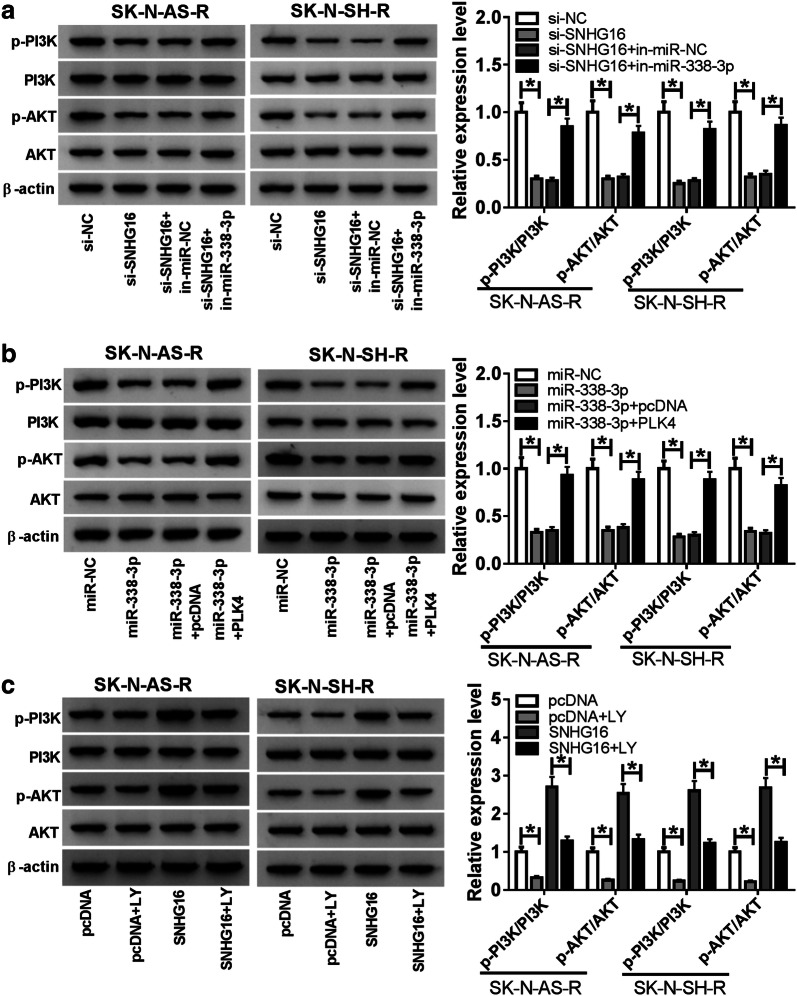
SNHG16/miR-338-3p/PLK4 axis can regulate the activation of PI3K/AKT pathway in neuroblastoma cells. **a**–**c** Protein expression levels of PI3K/AKT signaling pathway-related proteins in SK-N-AS-R and SK-N-SH-R cells from each group were detected by western blotting. The same experiment was repeated three times, and the average was taken. *P < 0.05

Western blot was conducted to measure the expression of PI3K/AKT pathway-related proteins (PI3K, p-PI3K, p-AKT and AKT). Results showed SNHG16 deletion reduced the levels of p-PI3K and p-AKT, while this reduction was rescued by the inhibition of miR-338-3p in SK-N-AS-R and SK-N-SH-R cells (Fig. 7a). Moreover, overexpressed PLK4 also abated miR-338-3p restoration induced decrease of p-PI3K and p-AKT expression levels in SK-N-AS-R and SK-N-SH-R cells (Fig. 7b). Subsequently, LY294002 (LY), the inhibitor of PI3K/AKT pathway, was used and results showed the introduction of LY inhibited the activation of PI3K/AKT pathway; moreover, LY treatment also attenuated SNHG16 overexpression-induced activation of PI3K/AKT pathway in SK-N-AS-R and SK-N-SH-R cells (Fig. 7c). Thus, these data indicated SNHG16/miR-338-3p/PLK4 axis could activate PI3K/AKT pathway in cisplatin-resistant neuroblastoma cells.

### SNHG16 deletion contributes to cisplatin-mediated inhibition on neuroblastoma tumor growth in vivo

**Fig. 8:**
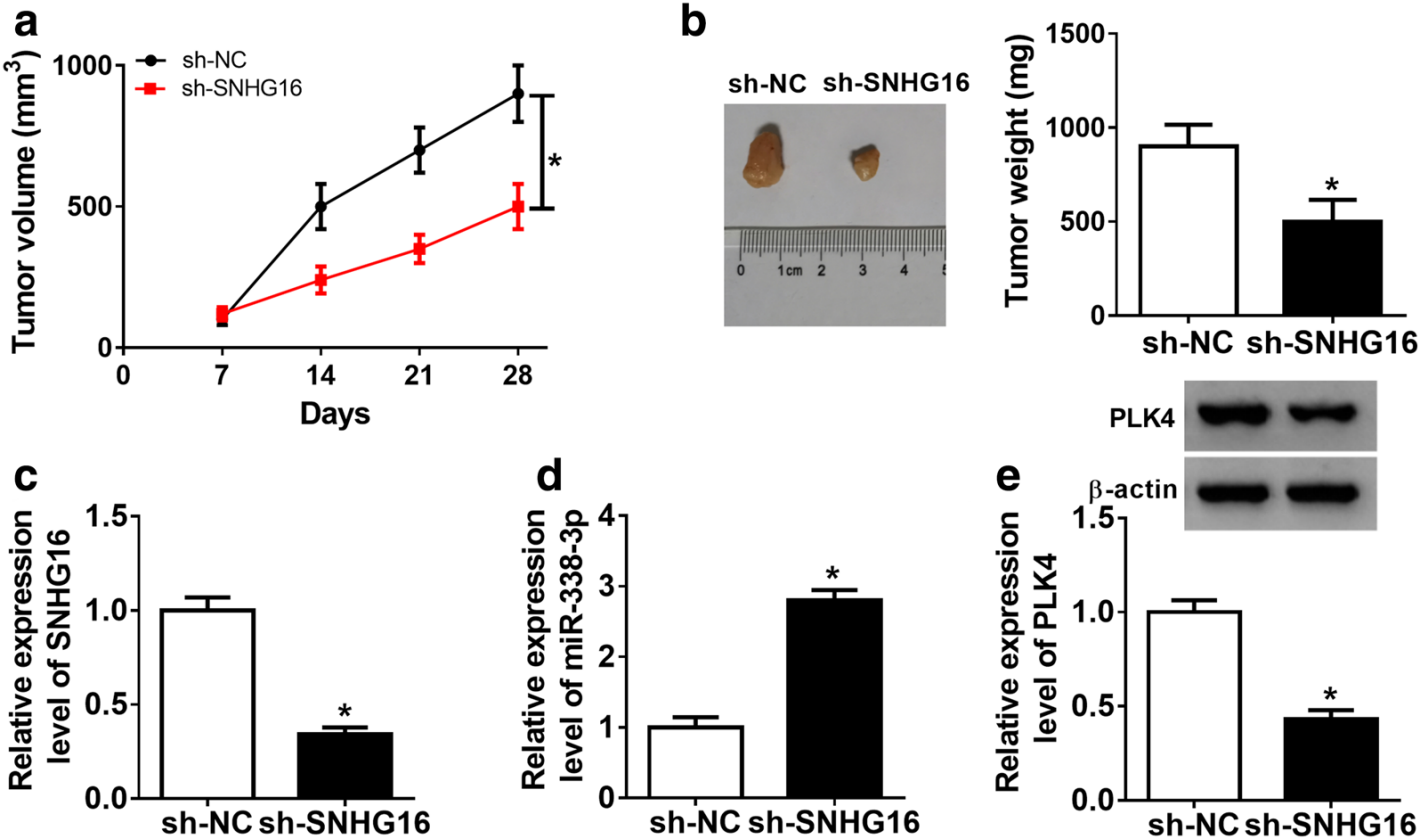
SNHG16 deletion contributes to cisplatin-mediated inhibition on tumor growth in vivo. SK-N-AS-R resistant cells stably infected with lenti-sh-SNHG16 or lenti-sh-NC were subcutaneously inoculated into the flanks of the nude mice, followed by intravenously injection with cisplatin (3 mg/kg) every 7 days. **a** Tumor volume was calculated every 7 days. **b** Mice were killed on day 28 after infection, and then tumor masses were excised and weighed. **c**–**e** The expressions of SNHG16, miR-338-3p, and PLK4 in excised tumor masses were determined using qRT-PCR and western blot, respectively (N = 3). N means that triplicate individual experiments were performed, and the average was taken. *P < 0.05

To explore the effects of SNHG16 on cisplatin-mediated tumor growth in vivo, SK-N-AS-R resistant cells stably infected with lenti-sh-SNHG16 or lenti-sh-NC were subcutaneously inoculated into the flanks of the nude mice, followed by intravenously injection with cisplatin (3 mg/kg) once per week after inoculation for 7 days. Subsequently, we found knockdown of SNHG16 significantly reinforced cisplatin-induced inhibition on the tumor growth, demonstrated by the decreased tumor volume and lowered tumor weight in sh-SNHG16-transfected SK-N-AS-R cells group (Fig. 8a, b). Furthermore, molecular analyses indicated SNHG16 deletion inhibited the expression of SNHG16 and PLK4, but promoted the level of miR-338-3p in excised tumor masses compared with the sh-NC group (Fig. 8c–e). Collectively, all these results implicated SNHG16 deletion exhibited a synergic effect with cisplatin in repressing neuroblastoma tumor growth in vivo.

## Discussion

Neuroblastoma is a malignancy arising from the improper differentiation of the embryonic sympathoadrenal lineage of the neural crest, leading to abnormal development of the paravertebral sympathetic ganglia and adrenal medulla in early childhood [4]. Cisplatin is a widely used and effective chemotherapeutic drug for patients with advanced or recurrent cancers in various types, including neuroblastoma [25]. Unfortunately, cisplatin resistance gradually occurs among neuroblastoma patients [26, 27]. Therefore, it is of great significance to investigate the molecular mechanisms underlying cisplatin resistance in neuroblastoma.

Increasing evidence has identified the involvement of lncRNAs in regulating cisplatin resistance in cancers [28, 29]. Among these lncRNAs, SNHG16 was revealed to enhance cisplatin resistance in osteosarcoma and hepatocellular carcinoma through regulating drug resistance phenotypes [12, 30]. However, it remains unclear whether SNHG16 contributes to cisplatin resistance in neuroblastoma. To address this question, we firstly examined the level of SNHG16 in cisplatin-resistant neuroblastoma tissues and cells, and a significant elevation of SNHG16 expression was found in cisplatin-resistant neuroblastoma tissues and cells. Immediately, functional experiments showed SNHG16 knockdown weakened cisplatin resistance and suppressed tumorigenesis in cisplatin-resistant neuroblastoma cells, reflected by the reduction of IC50 value to cisplatin, down-regulation of MRP-1 and P-gp protein levels, suppression of cell proliferation, migration and invasion, as well as enhancement of apoptosis in vitro. Furthermore, SNHG16 knockdown also enhanced the cytotoxicity of cisplatin in tumor growth in vivo.

Previous observations of macromolecular interactions have shown that lncRNAs contain multiple miRNA-binding sites and function as a competing endogenous RNAs (ceRNAs) for miRNAs to regulate the expression of target mRNAs or signaling pathway [31, 32]. The lncRNA-miRNA-mRNA regulatory network plays critical roles in the functional balance, which changes in each can trigger a series of pathological processes in cancers [33]. In this study, miR-338-3p was confirmed to be a target of SNHG16. MiR-338-3p is a well-recognized tumor suppressor and has been revealed to regulate cell drug resistance in colon cancer and hepatocellular carcinoma, which may be a promising candidate to boost the effectiveness of chemotherapy for cancers [34, 35]. This study demonstrated miR-338-3p was down-regulated in cisplatin-resistant neuroblastoma tissues and cells. After that, re-expression of miR-338-3p impeded cell malignant biological behavior and enhanced the sensitivity of cells to cisplatin in neuroblastoma. Besides that, we also found silencing miR-338-3p attenuated the action of si-SNHG16 on cisplatin resistance impairment and tumorigenesis inhibition in cisplatin-resistant neuroblastoma cells.

Immediately, we further explored the potential molecular mechanisms by which SNHG16/miR-338-3p axis affecting cell cisplatin-resistant and carcinogenesis in neuroblastoma, and we found that PLK4 was a target of miR-338-3p. PLK4 is an important regulator in the duplication of centriole, and its changes triggers carcinogenesis [23]. Additionally, PLK4 was also proved to mediate drug resistance in cancers [36, 37]. Many anticancer agents induce NF-κB nuclear translocation, excessive and prolonged activation of NF-κB has been established as a principal mechanism of tumor chemoresistance [38, 39]. PLK4 was found to phosphorylate IKBKE (inhibitor Of Nuclear Factor Kappa B Kinase Subunit Epsilon), and then activated NF-κB transcriptional [40]. In addition, taxanes can impair microtubule functions to kill cancer cells or inhibit the proliferation of cancer cells, γ-Tubulin is believed to participate in microtubule nucleation by forming a multiprotein ring complex (γ-TuRC), and PLK4 is an upstream regulator for γ-tubulin, which is dependent on format of the γ-tubulin-containing structure, suggesting the possible involvement of PLK4 in chemo-resistance [41]. Thus, PLK4 may be a potential regulator for drug resistance. In this study, we validated SNHG16 positively modulated PLK4 expression by sponging miR-338-3p in cisplatin-resistant neuroblastoma cells. Furthermore, PLK4 overexpression reversed the inhibitory effects of miR-338-3p on cisplatin resistance and malignant phenotype in neuroblastoma. Therefore, a SNHG16/miR-338-3p/PLK4 regulatory network was identified in neuroblastoma.

In the present study, we also discovered SNHG16/miR-338-3p/PLK4 axis could regulate the activation of PI3K/AKT pathway in neuroblastoma cells. The PI3K/AKT pathway is an intracellular signaling pathway and regulates multiple cellular processes in different cell types [42]. PI3K/AKT pathway contributes to the resistant phenotype and involves in the development of treatment strategies that target these specific signaling molecules or their downstream effectors [43].

However, there are still some limitations. Firstly, the data presented are based on a limited number of cell or animal experiments. Secondly, the role of PI3K/AKT pathway in cisplatin resistance in neuroblastoma remains vague. Thirdly, detailed function of SNHG16 on drug resistance phenotype is still unclear. Regarding the shortcomings of the present study, a larger cohort of the disease is necessary to validate these conclusion, and new study should be conducted to illustrate the function of PI3K/AKT pathway in cisplatin-resistant, as well as focus on how SNHG16 regulates drug resistance phenotype in vivo and in vitro.

## Conclusion

In conclusion, our studies demonstrated that SNHG16 contributed to cell cisplatin resistance and tumorigenesis in neuroblastoma via regulating miR-338-3p/PLK4 axis, suggesting a therapeutic target against cisplatin resistance in neuroblastoma.

## Supplementary information

**Additional file 1: Fig. S1.** The effects of SNHG16/miR-338-3p/PLK4 axis on the expression levels of PCNA, E-cadherin and N-cadherin. (A-B) Protein expression levels of PCNA, E-cadherin and N-cadherin in SK-N-AS-R and SK-N-SH-R cells from each group were detected by western blotting. The same experiment was repeated three times, and the average was taken. *P < 0.05.

## Data Availability

The data sets used and/or analyzed during the current study are available from the corresponding author on reasonable request.

## Abbreviations

MRP1: Multidrug resistance protein 1
lncRNAs: Long noncoding RNAs
miRNAs: MicroRNAs
PLK4: Polo like kinase 4
shRNA: Short hairpin RNA
PLK4: Polo like kinase 4
miR-338-3p: miR-338-3p mimic
in-miR-338-3p: miR-338-3p inhibitor
siRNA: Small interfering RNA
si-NC: siRNA negative control
qRT-PCR: Quantitative real-time polymerase chain reaction
cDNA: Complementary DNA
CCK-8: Cell counting kit-8
RIP: RNA immunoprecipitation
