# Supplementary figures and images for: SNHG16 promotes tumorigenesis and cisplatin resistance by regulating miR-338-3p/PLK4 pathway in neuroblastoma cells
Source: Cancer Cell Int. 2020 Jun 12;20:236. doi: 10.1186/s12935-020-01291-y (PMC7291484; doi:10.1186/s12935-020-01291-y)

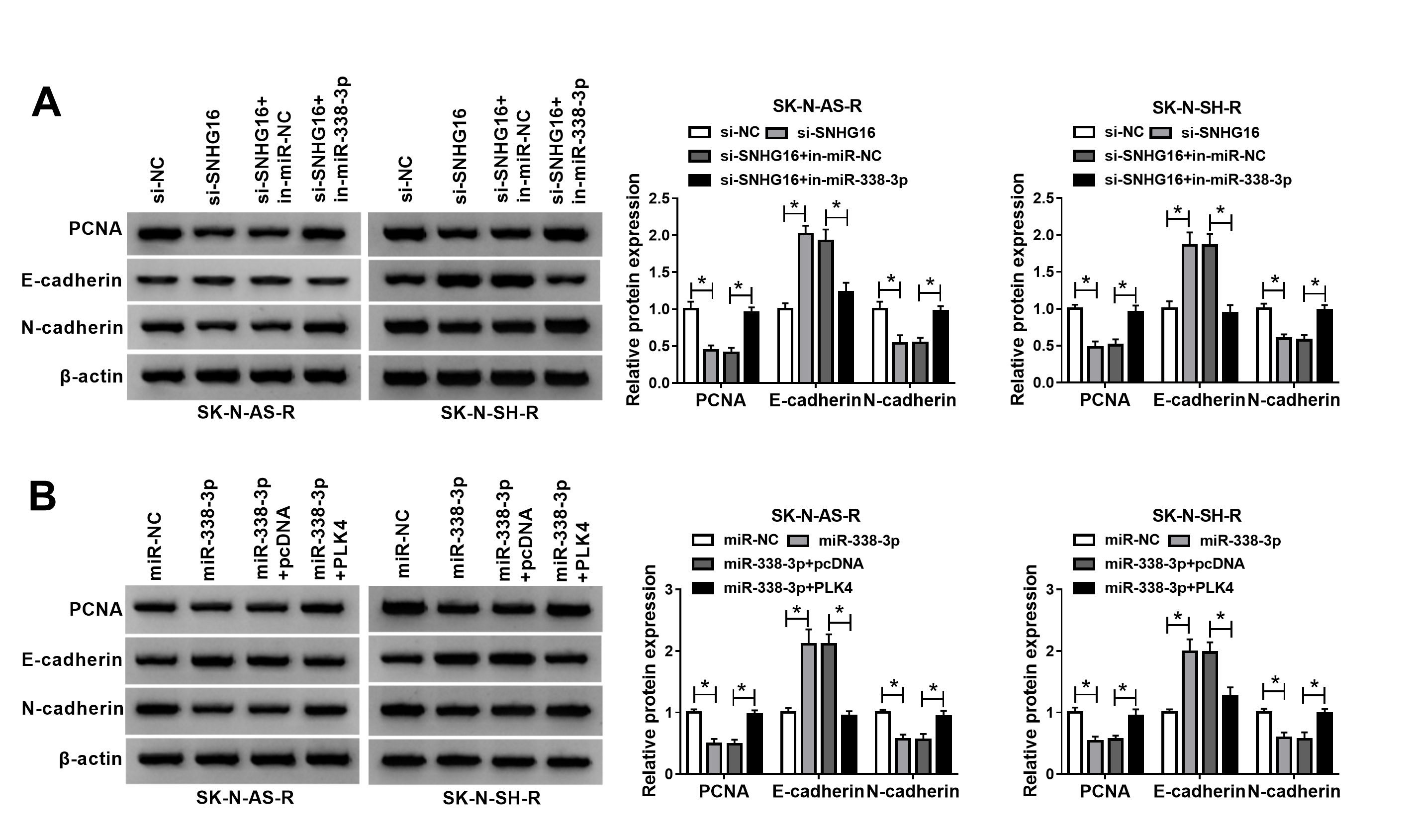

Supplement: Supplementary file 1 — Additional file 1: Fig. S1. The effects of SNHG16/miR-338-3p/PLK4 axis on the expression levels of PCNA, E-cadherin and N-cadherin. (A-B) Protein expression levels of PCNA, E-cadherin and N-cadherin in SK-N-AS-R and SK-N-SH-R cells from each group were detected by western blotting. The same experiment was repeated three times, and the average was taken. *P < 0.05. [file 12935_2020_1291_MOESM1_ESM.tif]
